# MicroRNA-431 regulates axon regeneration in mature sensory neurons by targeting the Wnt antagonist *Kremen1*

**DOI:** 10.3389/fnmol.2013.00035

**Published:** 2013-10-24

**Authors:** Di Wu, Alexander K. Murashov

**Affiliations:** ^1^Department of Neurobiology and Anatomy, Drexel University College of MedicinePhiladelphia, PA, USA; ^2^The Harriet and John Wooten Laboratory for Alzheimer’s and Neurodegenerative Diseases Research, Department of Physiology, School of Medicine, East Carolina UniversityGreenville, NC, USA

**Keywords:** miRNA, axon, regeneration, Wnt, *Kremen1*, miR-431, sensory neurons

## Abstract

MicroRNAs (miRNAs) are small, non-coding RNAs that function as key post-transcriptional regulators in neural development, brain function, and neurological diseases. Growing evidence indicates that miRNAs are also important mediators of nerve regeneration, however, the affected signaling mechanisms are not clearly understood. In the present study, we show that nerve injury-induced miR-431 stimulates regenerative axon growth by silencing *Kremen1*, an antagonist of Wnt/beta-catenin signaling. Both the gain-of-function of miR-431 and knockdown of *Kremen1* significantly enhance axon outgrowth in murine dorsal root ganglion neuronal cultures. Using cross-linking with AGO-2 immunoprecipitation, and 3′-untranslated region (UTR) luciferase reporter assay we demonstrate miR-431 direct interaction on the 3′-UTR of *Kremen1* mRNA. Together, our results identify miR-431 as an important regulator of axonal regeneration and a promising therapeutic target.

## INTRODUCTION

Axon loss is the hallmark of traumatic brain and spinal cord injury (SCI) as well as many neurodegenerative diseases including Alzheimer’s (Coleman and Perry, 2002). A body of research is focused on understanding the mechanisms of axon degeneration and promoting axon regeneration, however, the molecular mechanisms of neural repair remain poorly understood (Fang and Bonini, 2012). Growing evidence indicates that microRNA (miRNA) pathway controls regulatory mechanism involved in neural repair and regeneration (Strickland et al., 2011; Wu et al., 2011, 2012; Yu et al., 2011a; Zhang et al., 2011; Zhou et al., 2012). miRNAs are short, non-coding RNAs that silence gene expression by imperfect binding to 3′-untranslated region (UTR) of mRNA (Bartel, 2004; Filipowicz et al., 2008). miRNAs ability to simultaneously regulate the expression of several genes suggests that they are critical regulators of complex transcriptional networks (McNeill and Van Vactor, 2012). In the nervous system, miRNAs have been implicated in neurodevelopment (Smith et al., 2010), neurogenesis (Shi et al., 2010), and neurological disorders (Hebert and De Strooper, 2007; Kim et al., 2007). Recent observations have identified a group of miRNAs which reside within the distal axonal domain of superior cervical ganglia neuron suggesting miRNA role in the maintenance of axonal structure and function (Natera-Naranjo et al., 2010). In addition, several miRNAs have been associated with axon regeneration in peripheral nervous system (PNS) neurons (Strickland et al., 2011; Yu et al., 2011a; Zhang et al., 2011; Zhou et al., 2012) and axon development in cortical neurons (Dajas-Bailador et al., 2012).

Recent studies from our laboratory have demonstrated that ablation of Dicer, a key enzyme required for miRNA biogenesis, markedly impairs the regenerative axon growth *in vivo* and *in vitro*, indicating that the intact Dicer-dependent miRNA pathway is critical for successful peripheral nerve regeneration (Wu et al., 2012). In the current study, we examine the mechanism of miRNA action in axon regeneration. Here we show that injury-induced miR-431 stimulates regenerative axon growth by silencing *Kremen1*, a negative regulator of Wnt/beta-catenin signaling pathway. Both the gain-of-function of miR-431 and loss-of-function of *Kremen1* significantly enhance regenerative axon growth in dissociated dorsal root ganglia (DRG) neuronal cultures. Using cross-linking with AGO-2 immunoprecipitation (CLIP), and 3′-UTR luciferase assay we demonstrate miR-431 direct interaction on the 3′-UTR of *Kremen1* mRNA. Collectively, our observations provide the first evidence for a role of miRNA in regulating Wnt/beta-catenin signaling pathway in nerve regeneration and identify miR-431 as an important regulator and a potential therapeutic target.

## MATERIALS AND METHODS

### ANIMALS

Eight-week-old CD-1 male mice were obtained from Charles River laboratories (Wilmington, MA, USA). The animal use protocol was approved by the institutional Animal Care and Use Committee of East Carolina University, an Association for Assessment and Accreditation of Laboratory Animal Care-accredited facility. Animals were housed individually under standard laboratory conditions, with a 12 h light/dark schedule and unlimited access to food and water.

### CONDITIONING NERVE LESION

Before surgery, anesthesia was induced using an intraperitoneal ketamine (18 mg/ml)-xylazine (2 mg/ml) mixture (0.05 ml/10 g of body weight). The procedure followed a protocol described previously (Islamov et al., 2004). Exposure of the right sciatic nerve was performed with sterile surgical instruments through an incision on the middle thigh of the right hind limb. Approximately 5 mm of nerve was exposed from the sciatic notch to the trifurcation of the nerve. The exposed sciatic nerve was crushed in the mid-thigh for 15 s with a fine hemostat. The wounds were closed with 3M^TM^ Vetbond^TM^ Tissue Adhesive (3M, Saint Paul, MN, USA) and the animals were left to recover for 5 days.

### DISSOCIATED DRG CULTURES

Mouse L4/5 DRG neurons were collected 5 days after a conditioning sciatic nerve crush from both the intact side and injured side. DRGs were dissociated with collagenase and 0.25% trypsin in Dulbecco’s modified Eagle’s medium (DMEM; Invitrogen, Carlsbad, CA, USA). The dissociated DRGs were plated on poly-L-lysine and laminin (Invitrogen), coated plates. DRGs were grown in DMEM/F12 containing 10% horse serum, L-glutamine, and N2 supplement (Gemini Bio-product, West Sacramento, CA, USA) at 37°C for 18 h. To inhibit glial cell growth cytosin β-D-arabinofuranoside (Arac, 10 μM) and 5,6-dichlorobenzimidazole riboside (DRB, 80 μM; Sigma, Saint Louis, MO, USA) or 50 nM 5-fluoro-2′-deoxyuridine (Sigma) were added to the growth medium.

### PC12 CELL CULTURES

PC12 cells were cultured in DMEM containing 10% horse serum, 5% fetal bovine serum 2 mM glutamine, and penicillin and streptomycin (100 unitl/ml). The cells were plated on collagen-coated cell culture dishes. For nerve growth factor (NGF)-induced differentiation of PC12 cells, NGF (50 ng/ml) was added to cell culture medium to initiate neurite outgrowth. Medium was refreshed every 2–3 days.

### TRANSFECTION OF miRNA MIMICS AND INHIBITORS

In order to determine the biological effects of each individual miRNA on regenerative axon growth, we performed functional analyses for injury-induced miRNAs. Gain-of-function experiments were performed with Ambion^®^ Pre-miR^TM^ miRNA Precursor Molecules (Ambion, Austin, TX, USA), which are also called miRNA mimics. With transfection reagent, these small, chemically modified double-stranded RNA molecules can be introduced into cells and be taken up into the RNA-induced silencing complex (RISC), mimicking endogenous mature miRNAs activity. Loss-of-function analyses were performed with Ambion^®^ Anti-miR^TM^ miRNA inhibitors. The miRNA inhibitors are chemically modified, single-stranded nucleic acids designed to specifically bind to complementary miRNAs. The binding between endogenous miRNA and miRNA inhibitors down-regulates endogenous miRNAs activity.

All miRNA mimics and miRNA inhibitors were obtained from Ambion. Transient transfections of DRGs were performed using Lipofectamine^TM^ LTX and Plus Reagent (Invitrogen) according to the manufacturer’s protocol. To extend the time window for effective transfection of miRNA precursors and inhibitors, as well as, initiation of miRNA machinery, we incubated DRG neurons with 20 μM of SP600125 for the first 24 h according to a protocol previously described (Davare et al., 2009). SP600125 is a specific inhibitor of JNK and reversibly inhibits axonogenesis (Davare et al., 2009). We then released the block on axonogenesis from the SP600125 by washing out SP600125 and change culture media. DRG neurons were then cultured for an additional 24 h to allow axon formation.

### IMMUNOFLUORESCENT STAINING AND IMAGE ANALYSIS

The cells cultured on coverslips were fixed with 4% paraformaldehyde for 5 min and washed with phosphate buffered saline with Tween (PBST). After blocking with 10% goat serum for 1 h at room temperature, the samples were incubated with the indicated primary antibodies diluted at optimized concentrations at 4°C overnight. This was followed by incubation with secondary antibodies conjugated with FITC-, TX Red-, or Alexa Fluor^®^ (Invitrogen). Negative controls included samples processed in parallel with non-immune serum or without primary antibodies. After mounting the slides with anti-fading media (Invitrogen), images were viewed with an Olympus IX81 fluorescent microscope and captured with CellSens Dimension software (Olympus America Inc., Center Valley, PA, USA). The images we acquired were all single plane fluorescent images.

Quantification of axon length and measurement of axon branches were performed following previously described lab protocol (Murashov et al., 2005). For each coverslip, 30 images were taken, and from each, 10–15 neurons, which were completely distinguishable from neighboring cells, were chosen for further analysis. The axon length was quantified by tracing the image of neurites with the ImageJ software (NIH, Bethesda, MD, USA). The longest axon for each neuron was measured and recorded. The number of neurite branches per neuron was also determined from each neuronal population manually. Only primary branches, which are routinely defined as neurites originating from the neuronal soma and are at least longer than two times the diameter of the cell body were counted (Liu et al., 2002).

### IMMUNOBLOTTING ANALYSIS

Tissue samples were homogenized in ice-cold homogenization buffer (20 mM Tris, 2 mM EGTA, 2 mM EDTA, 6 mM β-mercaptoethanol, 1mM PMSF, and 10% Triton) containing protease inhibitor cocktail (Sigma), and centrifuged at 10,000 *g* for 10 min at 4°C. The supernatants were collected in fresh tubes and stored at -20°C. Proteins concentrations were quantified using the Bio-Rad reagent (Bio-Rad, Hercules, CA, USA) and samples for western blot analysis were prepared by boiling with standard stop buffer for 5 min. Equal amounts of solubilized proteins were loaded per lane on sodium dodecyl sulfate gels and separated by electrophoresis. The separated proteins were then transferred to immobilonP membranes (Millipore Corporation, Bedford, MA, USA).

Membranes were blocked in Odyssey blocking buffer (LI-COR, NE, USA) for 1 h at room temperature on a shaker, and then probed with a primary antibody in Odyssey blocking buffer at 4°C overnight. The membranes were washed three times with PBST, and then incubated with IRDye^®^ conjugated secondary antibodies for 1 h at room temperature with gentle shaking. The fluorescent signals on membrane were detected with the Odyssey^®^ Infrared Imaging System (LI-COR). Densitometry values were normalized to α-tubulin, to obtain the relative signal intensity.

### LIST OF ANTIBODIES

#### Primary antibodies

Mouse monoclonal neuro-specific β III tubulin antibody (TUJ-1) Covance Research Products, Inc. (Denver, PA, USA). Goat polyclonal antibodies against *Kremen1* (R&D Systems, Minneapolis, MN, USA). Rabbit anti-GAP-43 polyclonal antibodies (Millipore, Billerica, MA, USA). Mouse monoclonal anti-α-tubulin antibodies Zymed (Zymed Laboratories, Carlsbad, CA, USA).

#### Secondary antibodies

IRDye 800CW goat anti-Mouse IgG, IRDye 680LT goat anti-Rabbit IgG, and IRDye 800CW donkey anti-goat IgG secondary antibodies (LI-COR Corporate, NE, USA). For fluorescence studies, secondary FITC-, TX Red-conjugated IgG (Jackson ImmunoResearch Laboratories, Inc., West Grove, PA, USA) or Alexa Fluor 594 donkey anti-goat from Invitrogen were applied.

#### Cross-linked immunoprecipitation (CLIP) analysis

Argonaute CLIP method to identify *in vivo* targets of miRNAs followed procedure described previously (Jaskiewicz et al., 2012). DRG neuronal cell cultures were transfected with 100 nM of miR-431 mimic or a scrambled miRNA mimic negative control. Two days post-transfection, the cells were rinsed once in PBS and then placed in UVP CL-1000 cross-linker (UVP, Upland, CA, USA) with the cover off. Cells were irradiated once for 400 mJ/cm^2^ and once more for 200 mJ/cm^2^ to establish protein-RNA reversible cross-linking. Cells were lysed in cell lysis buffer (100 mM KCl, 5 mM MgCl_2_, 10 mM HEPES, PH 7.0, 0.5% NP-40, 1 mM DTT, 100 U/ml RNasin RNase inhibitor (Promega), 2 mM vanadylribonucleoside complexes solution (Sigma)) supplemented with a mixture of protease inhibitors (Invitrogen). Cells were then detached with a cell scraper and lysate was transferred to a tube on ice. Cell lysates were centrifuged at 16,000 *g* for 15 min at 4°C and the supernatants (the protein lysates) were transferred to sterile tubes for further immunoprecipitation. Prior to the immunoprecipitation, protein G agarose beads (Sigma) were equilibrated by washing twice with a wash buffer (0.5% NP-40, 150 mM NaCl, 2 mM MgCl_2_, 2 mM CaCl_2_, 20 mM Tris, pH 7.5, 5 mM DTT, with protease inhibitor) containing 1 mg/ml yeast tRNA and 1 mg/ml BSA. After pre-clearing the protein lysate with equilibrated protein G-agarose beads, 5 μl of each sample was saved as an input fraction. The protein lysate was immunoprecipitated with specific mouse monoclonal antibodies against Ago-2 (Wako, Richmond, VA, USA) or control serum and bounded by protein G agarose beads with agitation at 4°C overnight. After precipitation, the beads were washed three times with washing buffer. Afterward, the bonds between RNA and protein were disrupted by heating at 50°C for 30 min. RNA was then extracted and purified using Trizol (Invitrogen) and used for qRT-PCR.

#### Luciferase assays

Luciferase assays were performed using the pMIR-REPORT^TM^ miRNA expression reporter vector system (Ambion). pMIR-REPORT firefly luciferase (FL) plasmids were purified with Miniprep kit (Qiagen, Valencia, CA, USA) and digested with restriction enzymes *Spe*I and *Hind*III**(New England BioLabs, Ipswich, MA, USA). Linearized vectors from the restriction digestion were retrieved by agarose gel electrophoresis and gel purification of DNA using Gel Extraction Kit (Omega Bio-Tek, Inc., Norcross, GA, USA). The 3′-UTR regions of mouse *Kremen1* gene were amplified from mouse *Kremen1* cDNA clones (Source Bioscience, Nottingham, UK). The primers were designed as: 5′-ATAACTAGTGCTCCGCTCCAAGCTCGAGTTTGC 3′ and 5′-GCGAAGCTTTCTCTTTTGTAAAAGTTAAGTACC 3′. Restriction enzyme sites for *Spe*I and *Hind*III**were introduced into the PCR product to facilitate directional cloning. The 3′-UTR of *Kremen1* was inserted into downstream of FL gene in the pMIR-REPORT vector with T4 ligase (New England BioLabs), and subsequently transformed in DH5α competent cells (Invitrogen). Luciferase assays were performed using the Dual-Luciferase assay kit (Promega). PC12 cells (40,000) were cultured and co-transfected in 24-well plates with 400 ng of FL reporter construct, 100 nM miR-431 mimics or mimic negative controls, and 40 ng of pRL-TK control vector encoding renilla luciferase (RL; Promega). The transfection was performed with Lipofectamine 2000. Forty hours after transfection, the cells were harvested in passive lysis buffer and firefly and RL activities were measured in a Turner Biosystems 20/20n Luminometer (Turner Biosystems, Sunnyvale, CA, USA). The luciferase data is expressed as a ratio of FL to RL to normalize for transfection variability between samples. Luciferase experiments were repeated at least three independent times in triplicate.

#### miRNA and gene expression array analyses for DRG RNA

Total RNA for the microarray expression analysis was isolated from L4 to L5 DRGs, pooled from 10 mice at 4 days after sciatic nerve crush. Total RNA extraction was performed with miRVANA^TM^ miRNA isolation kit following the manufacturer’s instruction (Ambion). These pooled RNA samples were sent to UNC Lineberger Comprehensive Cancer Center Genomics Core for microarray analysis. After a quality control, they were hybridized to 8 × 15 miRNA one-color arrays (Agilent, Santa Clara, CA, USA). The same RNA samples were also hybridized to 4 × 44K mouse gene expression microarrays (Agilent) at the same Genomics Core. All microarray experiments were performed in duplicate and repeated twice. Normalization and further analyses of microarray data were performed with GeneSpring software (Agilent). Differentially expressed miRNAs were determined using a combination of t tests, with FDR correction of 0.1, and further defined by *p*-value < 0.05 after correction for multiple hypotheses. The analysis with GeneSpring allowed for identification of a different expression pattern of miRNAs in the crushed groups compared with the control groups. Statistically significant upregulated or down-regulated miRNAs were then selected for further analysis. All microarray data have been submitted to GEO (access number pending).

#### Real-time PCR (RT-PCR)

Total RNA was isolated from L4-L5 DRGs using *mir*Vana^TM^ miRNA Isolation Kit (Ambion). Total RNAs from DRG neuronal cell cultures were purified with RNAqueous Micro Scale RNA Isolation Kit (Ambion). RNA was quantified with a NanoDrop ND-1000 spectrophotometer (Thermo Scientific, Wilmington, DE, USA). Reverse transcription was performed with NCode^TM^ VILO^TM^ miRNA cDNA Synthesis Kit and SuperScript VILO cDNA Synthesis Kit (Invitrogen) for miRNA expression analysis and mRNA expression analysis, respectively. The real-time PCRs were carried out using EXPRESS SYBER^®^ GreenER^TM^ qPCR SuperMix Universal (Invitrogen) in triplicates for each cDNA sample on Applied Biosystems 7500 real-time PCR system (Applied Biosystems, Life Technologies, Carlsbad, CA, USA). Primers specific for each miRNA and mRNA were obtained from Invitrogen. As an internal control, primers for S12 (mitochondrial ribosome small subunit) were added for RNA template normalization, and the relative quantification of gene and miRNA expressions were calculated against S12 using a 2^-^^ΔΔ^^CT^ method. We routinely use S12 for qPCR studying axonal injuries. Other standard controls like beta-actin and GAPDH usually change in response to crush injury. All experiments were carried out three times independently.

#### List of primers

miR-21: 5′-TAGCTTATCAGACTGATGTTGA-3′

miR-431: 5′-CAGGCCGTCATGCAAA-3′

miR-744: 5′-GGGCTAGGGCTAACAGCA-3′

miR-124: 5′-GCGGTGAATGCCAAAAA-3′

miR-29a: 5′-TAGCACCATCTGAAATCGGTTA-3′

*Kremen1*: 5′-ACAGCCAACGGTGCAGATTAC-3′ and 5′-TGT TGTACGGATGCTGGAAAG-3′

GAP-43: 5′TGGTGTCAAGCCGGAAGATAA-3′ and 5′-GCTG GTGCATCACCCTTCT-3′

S-12: 5′-TGGCCCGGCCTTCTTTATG-3′ and 5′-CCTAAGCG GTGCATCTGGTT-3′

#### Statistical analysis

Data from multiple independent experiments were analyzed with GraphPad Prism version 5 for Windows (GraphPad Software, San Diego, CA, USA). The results were expressed as mean ± standard error of the mean in graphic and text representations. To determine the difference between three or more groups, a one-way analysis of variance (ANOVA) followed by Bonferroni’s multiple comparison tests was utilized. For the analysis of two independent groups, Student’s *t*-test was used. A *p*-value of less than 0.05 was considered statistically significant.

## RESULTS

### miRNAs ARE DIFFERENTIALLY EXPRESSED IN DRG UPON SCIATIC NERVE INJURY

**FIGURE 1:**
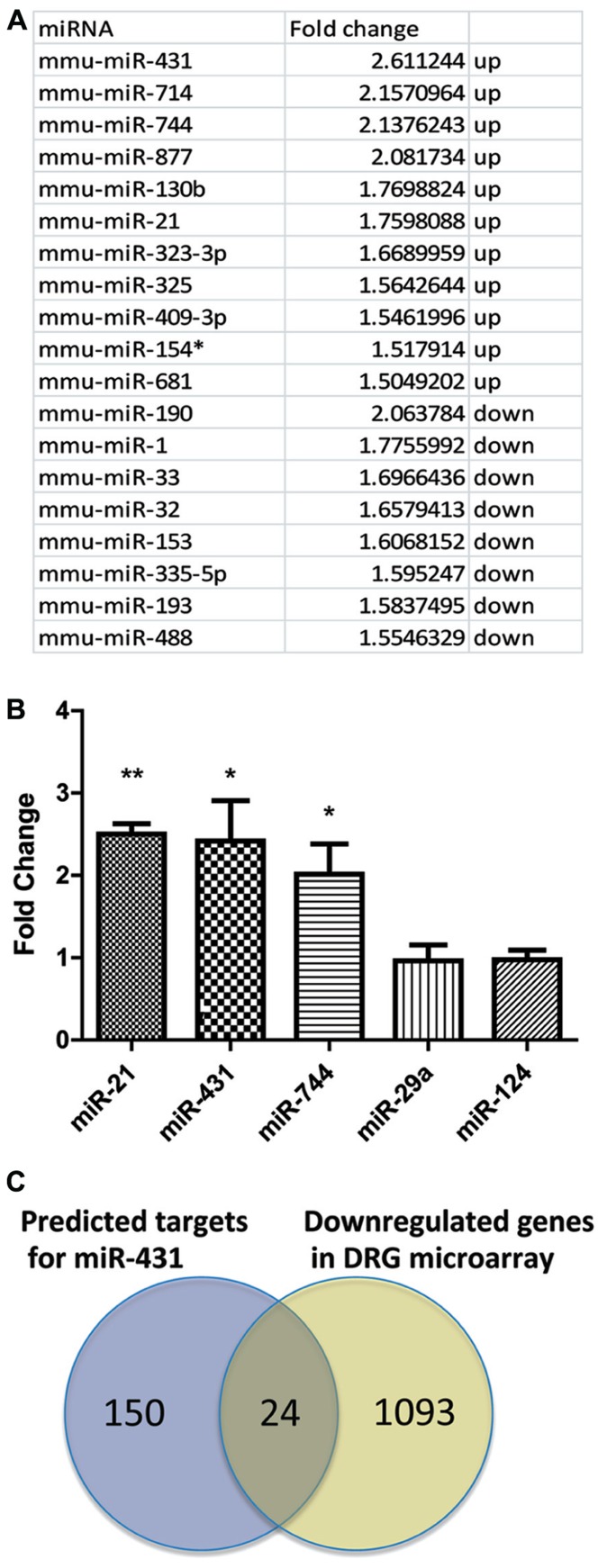
**Sciatic nerve injury induced changes in miRNA expression profile in DRG.** **(A)** Total RNA for the microarray expression analysis was isolated from DRG 4 days after sciatic nerve crush. Agilent arrays were done in duplicates and repeated twice. Normalization and analyses were performed with GeneSpring software. miRNAs with a statistically significant upregulation or down-regulation over 1.5-fold were listed in the table. **(B)** Three miRNAs that were significantly upregulated were selected for further validation. Real-time qPCR for miRNA validated the relative changes in miRNA level. miRNA expression was normalized to reference gene s12. The graph indicates a significant increase of miR-744, miR-431, and miR-21 in DRG after sciatic nerve crush, whereas the expression level of miR-124 and miR 29a did not change (*p < 0.05, **p < 0.01, *N* = 3). **(C)** Venn diagram of overlap in predicted miR-431 target genes and down-regulated genes in DRG after conditioning sciatic nerve lesion. The potential targets of miR-431 were chosen using three algorithms , , and . Down-regulated genes were selected using fold change cut-offs of >2 and significance *p*-values of <0.05 expression based on microarray data for DRGs 4 days post-sciatic nerve injury. Overlap shows 24 genes having predicted binding site for miR-431 and significantly down-regulated expression level in DRG microarray. A one-way ANOVA followed by Bonferroni’s multiple comparison tests was utilized. For the analysis of two independent groups, Student’s*t*-test was used.

We analyzed miRNA expressions in DRGs using microarrays at 4 days after sciatic nerve crush. DRGs were collected from both the pre-conditioned side, as well as the contralateral uninjured side. RNA from the contralateral uninjured side served as a control group. At 4 days post-injury, pre-conditioned DRG neurons show robust regenerative axon growth (Forman et al., 1980). RNA from the pre-conditioned DRG was considered the actively regenerating group. By comparing the miRNA expression pattern from pre-conditioned DRG and control DRG, miRNAs that were upregulated and down-regulated during the process of regeneration were determined. Several miRNAs demonstrated differential expression based on regenerative growth condition. Using 1.5-fold cut-off, statistical analyses revealed that 19 miRNA were differentially expressed in the pre-conditioned DRG compared to the non-conditioned contralateral DRG. Of those 19, 11 miRNAs had higher expression level in pre-conditioned group and the other eight miRNAs had lower expression level in DRG during regeneration (**Figure 1A**). miR-431, miR-714, miR-744, miR-877, miR-130b, miR-21, miR-323-3p, miR-325, miR-409-3p, miR-154*, and miR-681 were significantly increased 4 days post-sciatic nerve crush in pre-conditioned DRGs, while miR-190, miR-1, miR-33, miR-32, miR-153, miR-335-5p, miR-193, and miR-488 showed significantly decreased expression. The most upregulated miR-431 was selected for further analyses.

We validated the microarray data for miR-431 using real-time qPCR. We also included miR-744 and miR-21 as positive controls and miR-124 and miR-29a as non-regulated controls in our real-time PCR experiments. These last two miRNAs play various roles in neurodevelopment and maintenance of neuronal cell homeostasis (Cheng et al., 2009; Shioya et al., 2010); however, they did not show changes in their expression in our array data. In agreement with the microarray data, miR-431, miR-744, and miR-21 were significantly upregulated in regenerating neuronal cells. We detected 2.4-fold upregulation of miR-431, a twofold upregulation of miR-744, and a 2.5-fold upregulation of miR-21, respectively (**Figure 1B**). At the same time, RT-qPCR experiments showed that miR-29a and miR-124 did not change their expression during regeneration.

### GAIN-OF-FUNCTION OF miR-431 INCREASES REGENERATIVE OUTGROWTH

**FIGURE 2:**
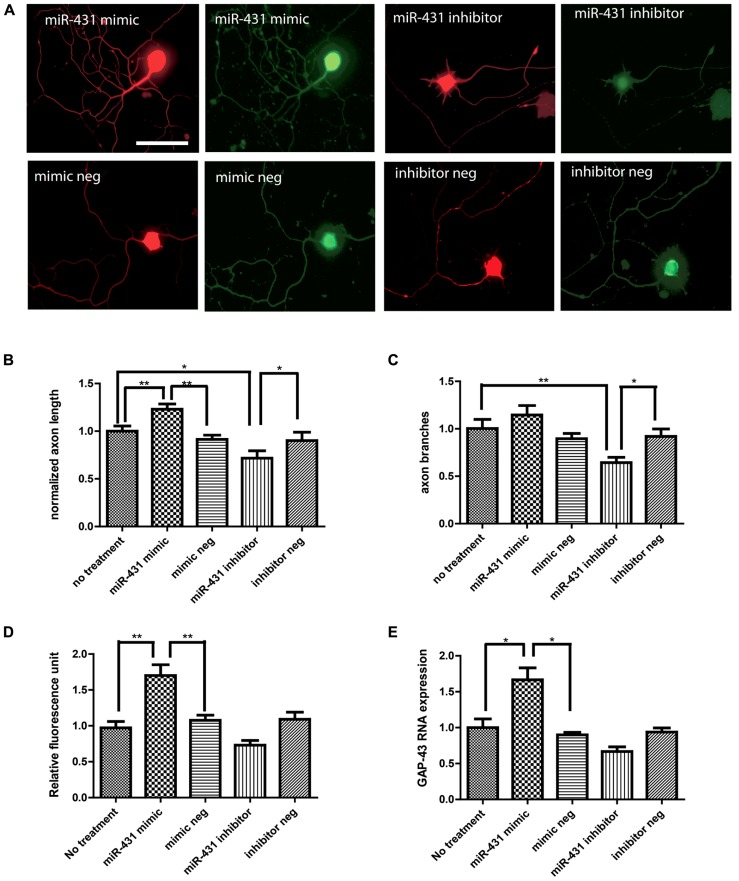
**miR-431 increases axon outgrowth in DRG neurons. Effects of miR-431 mimic and inhibitor on axon outgrowth.** **(A)** Left panel shows the effect of the transfection of DRG neurons with miR-431 mimic. Right panel depicts the effect of transection with miR-431 inhibitor. Negative controls for miR-431 mimic and inhibitor are indicated on the lower images. Cells were stained with primary antibodies against neuronal β-tubulin and signals were visualized with TX-Red conjugated secondary antibody (scale bar: 50 μm). The expression of GAP-43, a marker for axon regeneration, was detected using an anti-GAP-43 antibody and visualized with FITC-conjugated secondary antibodies. The effect of miR-431 on axon length **(B)** and on axon branching **(C)** was quantified. Overexpression of miR-431 significantly increased axon extension, whereas suppression of miR-431 significantly blocked axon branching. The fluorescence signal intensity against GAP-43 was quantified in **(D)**. The significant increase in GAP-43 immunofluorescence reflects increase in regenerative axon growth. **(E)** Significant increase in GAP-43 expression on mRNA level quantified by RT-qPCR (*p < 0.05, **p < 0.01, *N* = 50). A one-way ANOVA followed by Bonferroni’s multiple comparison tests was utilized.

To investigate the role of miR-431 in regenerative axon growth, we manipulated the level of miR-431 in dissociated DRG neurons. We observed a positive association between miR-431 expression and neurite outgrowth in dissociated DRG neuronal cell culture (**Figure 2A**). Increased mir-431 level was achieved by applying miR-431 mimic to DRG neuronal cell cultures at a final concentration of 100 nM. Overexpression of miR-431 significantly increased axon length. Additionally, blocking miR-431 activity with miR-431 inhibitor significantly inhibited neurite extension (no treatment control group: 100 ± 5%; miR-431 mimic group: 130 ± 6%; mimic negative group: 91 ± 4%; miR-431 inhibitor group: 75% ± 7%; inhibitor negative control: 90 ± 8%; **Figure 2B**). Moreover, manipulating miRNA-431 levels also affected axon branching, and led to a decrease in the number of branches per neuron due to transfection with miR-431 inhibitor (no treatment control group: 100 ± 9%; miR-431 mimic group: 110 ± 10%; mimic negative group: 82 ± 7%; miR-431 inhibitor group: 64% ± 6%; inhibitor negative control: 86 ± 10%; **Figure 2C**).

We next studied GAP-43 expression in DRG neurons with miR-431 mimic and inhibitor treatments, as a strong association between neurite outgrowth and expression of GAP-43 has been reported in previous studies (Benowitz and Routtenberg, 1997). We observed significant increase in GAP-43 immunostaining caused by transfection with miR-431 (**Figure 2D**). GAP-43 mRNA level was further studied with RT-qPCR. **Figure 2E** clearly demonstrates a significant increase in GAP-43 mRNA in the cultures treated with 100 nM of miR-431 mimics, as compared to the group treated with the scrambled miRNA mimic control. This relates to immunofluorescent data demonstrating significant increase in axon outgrowth after overexpression of miR-431.

### IDENTIFICATION OF miR-431 mRNA TARGETS

We used three databases^1^ to generate a list of mRNAs with potential binding site for miR-431 in their 3′-UTR. The potential candidates were further selected based on evaluation of the gene expression microarray data for DRGs 4 days post-sciatic nerve injury (SNI). We hypothesized that an increased expression of miR-431 in pre-conditioned DRG, would negatively associate with expression of the target mRNAs in the same RNA samples. Using GeneSpring 10 software package (Agilent) we performed joint analysis of miRNA and gene expression data. This allowed us to narrow the list of potential targets to 24 genes. These 24 genes met both criteria, of having a predicted binding site for miR-431 in their 3′-UTR and significantly down-regulated expression level in DRG microarray (**Figure 1C**).

To investigate which genes may be regulated by miR-431, we initially screened potential targets in neuronal PC12 cells overexpressing miR-431. Transient overexpression of miR-431 was achieved using transfection of PC12 cells with miR-431 mimic. The expression of potential targets was studied with real-time RT-qPCR. The experiments revealed that only six genes (*Braf, Eif2s2, Kremen1, Msi2, Tnrc6b, Zkscan1*) were significantly down-regulated by miR-431 in PC12 cells (**Table 1**). We then applied the same approach to test these six genes with overexpression of miR-431 in primary DRG neurons. In the RT-qPCR experiments, overexpression of miR-431 led to significant suppression of the expression of only three genes including *Braf*, *Kremen1*, and *Zkscan1* (**Table 1**). Based on the literature data indicating that *Kremen1* is an antagonist of Wnt signaling pathway (Nakamura and Matsumoto, 2008), which is critical for axonal remodeling (Purro et al., 2008), we focused our subsequent experiments on characterization of *Kremen1*–miR-431 interaction.

**Table 1:** Effect of miR-431 overexpression on levels of potential target genes in PC12 cells and primary DRG culture.

Gene	PC12 cells		DRG culture	
	Relative value	SEM	Relative value	SEM
*Braf*	**0.323**	**0.1245**	**0.5133**	**0.07055**
*Cwf1912*	5.833	0.8762		
*Dlst*	1.85	0.1531		
*Eif2s2*	**0.4367**	**0.06766**	0.8333	0.2028
*Fgf12*	0.68	0.1531		
*Hip1*	1.07	0.2608		
*Kremen1*	**0.3833**	**0.05364**	**0.6733**	**0.0393**
*Luc712*	0.69	0.1054		
*Msi2*	**0.4133**	**0.05364**	1.04	0.07024
*Ncam1*	1.103	0.2284		
*Nudcd3*	0.91	0.1002		
*Slc30a10*	1.163	0.02906		
*Son*	4.033	0.5044		
*Tcf712*	2.043	0.4937		
*Tnrc6b*	**0.58**	**0.0755**	0.9467	0.245
*Vezt*	1.253	0.1141		
*Wnk3*	0.9433	0.1601		
*Zeb2*	0.9733	0.1742		
*Zkscan1*	**0.2967**	**0.06009**	**0.45**	**0.06429**

*Transient overexpression of miR-431 was achieved using transfection with its mimic. Relative mRNA levels of the potential target genes were evaluated by real-time RT-qPCR. Bold numbers indicate significant decrease in gene expression. Only three genes *Kremen1*, *Braf*, and *Zkscan1* were significantly down-regulated in primary neuronal culture.*

**FIGURE 3:**
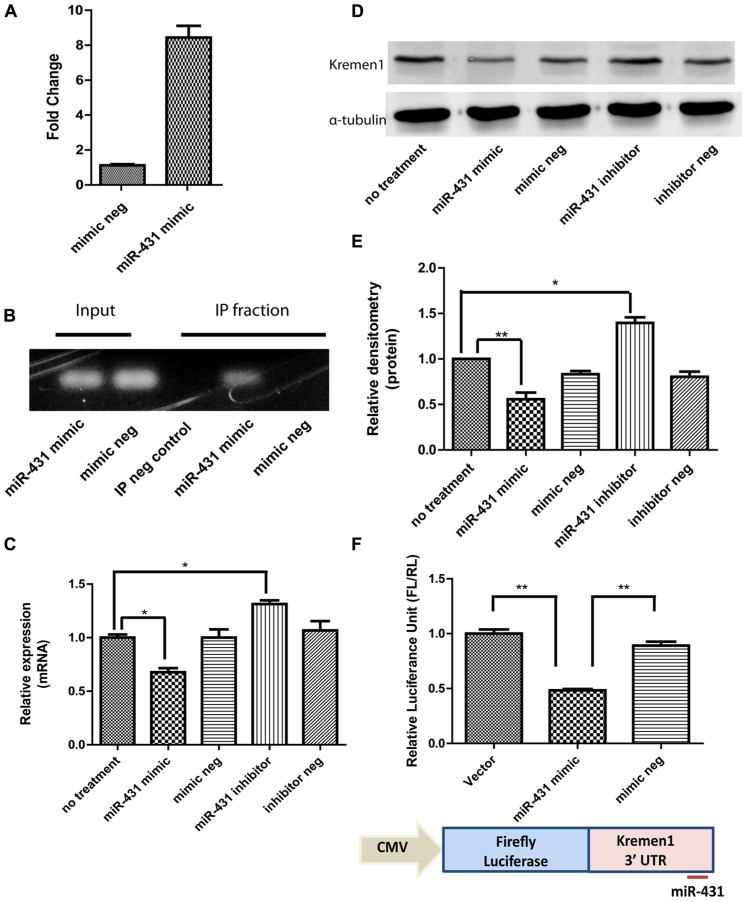
**miR-431 regulates *Kremen1* expression.** **(A)** RT-qPCR confirmed the increase of miR-431 level in DRG neuron after the transfection of miR-431 mimic. **(B)** Although overexpression of miR-431 decreased *Kremen1* mRNA in total cell lysates (input), it enhanced the binding between *Kremen1* mRNA and Ago-2 complex. In the Ago immunoprecipitated fractions, there was an increased amount of *Kremen1* mRNA. The lack of signal in the non-specific serum IP sample (IP neg. control) confirmed the specificity of the IP. **(C)** miR-431 negatively regulated *Kremen1* expression at mRNA level. Treatment of miR-431 mimics in DRG neuronal cultures significantly inhibited *Kremen1* expression as compared with that of control groups. On the contrary, suppression of miR-431 activity significantly enhanced the expression of *Kremen1* mRNA. **(D)** Western blot analysis of *Kremen1* expression exhibited similar negative correlation of miR-431 and *Kremen1* expression. Cells transfected with miR-431 mimics had decreased protein level of *Kremen1*, whiles cells transfected with miR-431 inhibitors had an increased expression of *Kremen1*. α-tubulin was used as the loading control and was used to normalize densitometry values. **(E)** The quantification of densitometric levels of *Kremen1*. **(F)** PC12 cells were transfected with *Kremen1* 3′UTR-firefly Luciferase constructs for luciferase assays. Co-transfection with miR-431 mimics significantly reduced the luciferase activity (*p < 0.05, **p < 0.01), whereas co-transfection with mimic negative controls did not affect the expression of firefly luciferase gene. A one-way ANOVA followed by Bonferroni’s multiple comparison tests was utilized.

To investigate a direct interaction between target mRNAs and miR-431 in RISC, CLIP of the Ago-2 protein, the central component of the RISC was carried out. Applying miR-431 mimic to DRG neurons increased the expression level of miR-431 ß7.75-fold in DRG neuronal cell cultures (**Figure 3A**). Electrophoresis of CLIP samples confirmed the miR-431 induced association of *Kremen1* mRNA with RISC, suggesting *Kremen1 *as the target gene for miR-431 (**Figure 3B**). **Figure 3B** shows the RT-PCR of *Kremen1 *mRNA presented in the total RNA (input) and IP fractions from DRG cultures treated with miRNA mimic and the mimic negative control. In the total RNA samples from DRG cultures, overexpression of miR-431 reduced the amount of stable *Kremen1 *mRNA when compared to the miRNA mimic negative control group. In the Ago-2 immunoprecipitated RNA samples, overexpression of miR-431 clearly increased the level of Ago-2 associated *Kremen1 *mRNA. In the IP negative control group (non-immune serum), no detectable *Kremen1 *mRNA was observed, confirming the specificity of the precipitation (**Figure 3B**).

### LUCIFERASES REPORTER ASSAY CONFIRMS miR-431 TARGET *Kremen*1 3′UTR

*Kremen1* has one binding site for miR-431 at its 3′-UTR, at the position 2530–2536 bp. It corresponds perfectly to nucleotides 2–7 of the mature miRNA in mouse, rat, and human. In addition, the seed target site is close the poly-A tail, which increases its accessibility. To confirm miR-431 direct interaction on *Kremen1* 3′ UTR, we established a *Kremen1* 3′UTR-FLs construct with the 3′-UTR of *Kremen1* inserted downstream of the FL gene. This construct allowed us to quantitatively evaluate the regulatory effect of miR-431 on the 3′-UTR of *Kremen1*. PC12 cells were transiently transfected with miR-431 mimics or mimic negative controls, *Kremen1 *3′UTR-FL construct, and RL plasmid DNA as internal control. As shown in **Figure 3F**, co-transfection of miR-431 mimic and *Kremen1 *3′UTR-FL construct resulted in significant decrease in FL activity. Luciferase activity reduced to 48% compared with the vector control, whereas co-transfection of mimic negative controls and *Kremen1 *3′UTR-FL construct did not affect the expression of FL gene (**Figure 3F**). Together, these data suggest that miR-431 actively modulates *Kremen1* protein and RNA expression within DRG neurons through association with *Kremen1* 3′UTR.

### miR-431 MODULATES *Kremen*1 EXPRESSION AT mRNA AND PROTEIN LEVELS IN PRIMARY NEURONAL CULTURES

To show that miR-431 regulates endogenous *Kremen1* in DRG neurons, we transfected cells with either miR-431 mimics, miR-431 inhibitors, mimic negative control, or inhibitor negative control. Since miRNA-mediated gene regulation can destabilize target mRNA and reduce the level of the target mRNA, we used RT-qPCR to determine the effect of miR-431 on *Kremen1*. We observed that transient transfection with miR-431 mimic, decreased the mRNA level of *Kremen1* to 30%. Application of miR-431 inhibitors significantly elevated the mRNA level of *Kremen1* (**Figure 3C**). These results demonstrated that miR-431 level is inversely correlated to *Kremen1* expression at mRNA level in DRG neurons.

We then performed proteomic analysis of *Kremen1* in DRG neurons. Whereas endogenous miR-431 was inhibited by transfection with miR-431 inhibitor, the expression level of the *Kremen1* protein was significantly higher than in control groups. Quantification of three independent experiments revealed that miR-431 reduced *Kremen1* protein levels by 50% when compared with the mimic negative control group. On the other hand, inhibition of endogenous miR-431 resulted in a significant increase of *Kremen1* expression by 45% (**Figures 3D,E**).

### *Kremen*1 EXPRESSION IN DRG *IN VIVO*

**FIGURE 4:**
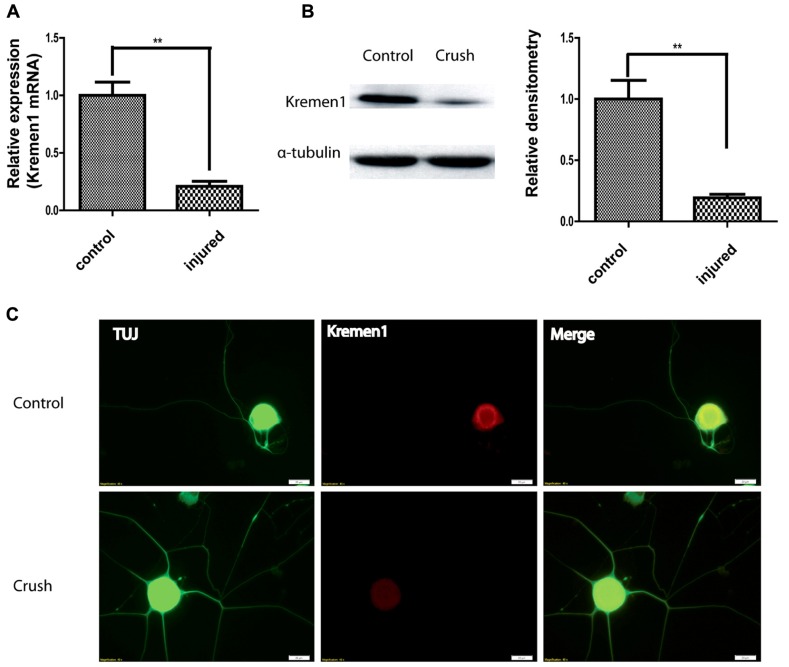
**Nerve crush injury reduces *Kremen1* expression.** **(A)** Total RNA was isolated from control or crush-injured mouse DRG, and relative expression of *Kremen1* was determined using RT-qPCR. GAPDH and S12 were used to normalize for RNA loading. **(B)** Western blot analysis of total DRG lysates at 4 days post-crush injury. α-Tubulin was shown as a loading control. As shown in the quantified densitometry data, there was a significant decrease of *Kremen1* expression during nerve regeneration. **(C)** Immunofluorescent staining in dissociated DRG neurons demonstrated the expression of *Kremen1* within neurons. *Kremen1* as a transmembrane receptor was shown to be located in cell bodies, but not axons. TUJ staining was used to visualize neuronal cells. Preconditioning of sciatic nerve clearly promotes regenerative axon growth in DRG neurons, and this phenomenon is accompanied by a decrease in *Kremen1* expression. Scale bar: 20 μm. (**p < 0.01) For the analysis of two independent groups, Student’s *t*-test was used.

After establishing a physical interaction between miR-431 and *Kremen1*, we next investigated the expression patterns of *Kremen1* during axon regeneration. From gene expression array data, *Kremen1* expression in DRG decreased at 4 days after SNI, suggesting its expression was down-regulated as the peripheral nerve regenerated. To further reveal physiological role miR-431 *Kremen1 *interaction, we analyzed expression of *Kremen1* at RNA and protein levels from control and regenerating DRGs. RT-qPCR revealed that *Kremen1* RNA expression decreased fourfold at 4 days after sciatic nerve crush, when axons exhibit robust regenerative growth (**Figure 4A**). Similarly, we found that *Kremen1* protein was reduced in DRGs at 4 days post-injury. The Western blot data showed a significant 80% decrease in *Kremen1* expression after SNI when compared to control (**Figure 4B**).

The expression of *Kremen1* in DRG neuron was further examined using indirect immunofluorescence (IIF). IIF with antibodies against *Kremen1* revealed the localization of *Kremen1* in dissociated DRG neurons. In both pre-conditioned and control groups, the immunoreactivity of *Kremen1* was detected mainly in neuronal cell bodies, however, there was less *Kremen1 *immunostaining in the group with sciatic nerve crush (**Figure 4C**). These data further support a functional relationship between miR-431 and *Kremen1* in regenerating DRG neurons and suggest a role of *Kremen1* in peripheral nerve regeneration.

### FUNCTIONAL ANALYSIS OF *Kremen*1 ROLE IN AXON REGENERATION

**FIGURE 5:**
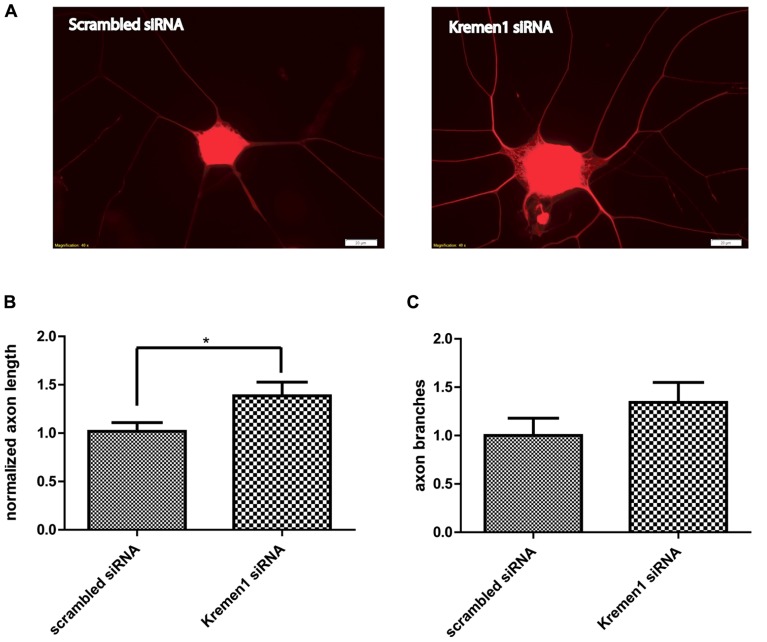
**Knockdown of *Kremen1* increases neurite outgrowth.** **(A)** Neurite outgrowth in *Kremen1* siRNA and scrambled siRNA treated DRG neurons was detected by TUJ immunostaining. Representative images show that *Kremen1* siRNA significantly decreased *Kremen1* expression level, which was accompanied by an increase of axon outgrowth. Scale bar: 20 μm. As the quantification performed in miR-431 functional analysis, we measured the length of the longest axon for each neuron **(B)** and counted the number of branches for each neuron **(C)**. Inhibition of *Kremen1* significantly increased the length of axon, however, its effect on neurite branching was not significant. *-*p* < 0.05. For the analysis of two independent groups, Student’s *t*-test was used. Scale bar: 20 μm.

Given the effects of miR-431 on *Kremen1* expression and the role of miR-431 in neurite outgrowth, we investigated the effect of *Kremen1* knockdown on regenerative axon growth. Two groups of DRG neurons were transfected with either siRNA specifically targeting *Kremen1* mRNA, or scrambled siRNA (negative control). The differences in the regenerative growth between *Kremen1 *siRNA group and control scrambled siRNA group were quantified based on axon elongation and branching. The experiments revealed that knockdown of *Kremen1* significantly increased axon length in dissociated DRG cultures (**Figure 5**). The axon length in the *Kremen1* knockdown group increased ~30% in comparison to the scrambled siRNA control group. This effect on axon outgrowth is similar to the effect of miR-431 overexpression on axon outgrowth reported earlier (**Figure 2B**). Taken together, these results indicate that miR-431 mediates increase of axon growth through *Kremen1* repression.

## DISCUSSION

### ALTERED miRNA EXPRESSION FOLLOWING NERVE INJURY

Our microarray experiments identified a group of injury-regulated miRNAs in DRG neurons after conditioning sciatic nerve lesion. Alterations in miRNAs have been recently shown in several studies profiling miRNA expression after nerve injuries in the central nervous system. Microarray based analysis of miRNA in the rat cerebral cortex after traumatic brain injury revealed that a set of miRNAs were differentially expressed at 6, 24, 48, and 72 h after injury. At all-time points post-injury, miR-21 was consistently highly expressed in the cerebral cortex (Lei et al., 2009). Changes in miRNA expression have also been studied by microarray analysis in hippocampus after traumatic brain injury. At three and 24 h after controlled cortical impact injury, 35 miRNA exhibited increased expression levels and 50 miRNA exhibited decreased expression level (Redell et al., 2009). Following a contusive SCI in adult rats (Liu et al., 2009), 60 miRNAs showed significant changes in their expression level in the injured spinal cord at 4 h, 1, and 7 days. Among those 60 miRNAs, 30 were upregulated, 16 were down-regulated, and 14 showed early upregulation at 4 h followed by down-regulation at 1 and 7 days post-SCI (Liu et al., 2009). Recently, observations on miRNA expression have been extended to the PNS. miRNA expression has been profiled following SNI in proximal stumps of injured sciatic nerve and DRG by microarray and deep sequencing in several studies (Strickland et al., 2011; Yu et al., 2011b; Zhou et al., 2012). Following sciatic nerve transection, 20 miRNA transcripts displayed a significant change in expression levels at 7-day post-axotomy in rat DRG (Strickland et al., 2011). Both miR-21 and miR-431 showed significant upregulation in DRG after SNI, comparably to our current data. Taken together, Strickland’s and our study, demonstrate that miR-21 and miR-431 are implicated in peripheral nerve regeneration across species. Strickland’s study further revealed that miR-21 promoted the regenerative growth of the injured neuron by targeting the Sprouty2 protein (SPRY2; Strickland et al., 2011).

In our studies, we focused on miR-431, which was the most upregulated miRNA in DRG microarray after nerve injury in our experiments. miR-431 was initially identified as central nervous system specific miRNA as it was cloned from brain tissue of mouse embryos (Wheeler et al., 2006). Whole mount *in situ* hybridization revealed miR-431 localization to the developing spinal cord and brain with particularly strong expression in the pons. The pons is particularly rich in synapses because ninety percent of the descending axons passing through the midbrain synapse on neurons in the pons (Wheeler et al., 2006). However, to date, limited information is available about miR-431 physiological function. Recent observation has linked expression of miR-431 to regulation of cell viability (Tanaka et al., 2012). miR-431 was upregulated by the addition of human fibroblast interferon (HuIFN-β) in a non-cancer HuIFN-β sensitive cell line RSa, with concomitant suppression of IGF1R signaling and reduction of cell viability (Tanaka et al., 2012). However, at this time, the function of miR-431 in the nervous system remains uncertain.

### THE FUNCTION OF miR-431 IN REGENERATIVE AXON GROWTH

To determine the role of miR-431 in axon regeneration, miR-431 gain- and loss-of-function were investigated in DRG neuronal cultures. Application of miR-431 mimics markedly increased the intracellular miR-431 level and promoted regenerative axon outgrowth. miR-431 gain-of-function correlated with longer axons, more branches, and higher GAP-43 expression, a marker of regeneration. In contrast, transfection of miR-431 inhibitors impaired the regenerative axon growth, as significantly shorter axons and fewer branches were observed in DRG cultures. Analyses of 24 putative targets of miR-431, showed that only six were suppressed in PC12 cells and even less genes were suppressed in DRG primary neurons. This could be related to the specificity of miR-431 to these genes, and to the fact that down-regulation of less specific targets is more easily detected in PC12 cells. The difference may be also related to the fact that the cells were from different species; PC12 were from rat and DRG culture was from mouse.

We have further identified *Kremen1* as the target that mediates the effects of miR-431 on neuronal cells. miR-431 expression inversely relates to *Kremen1*. The direct interaction between miR-431 and *Kremen1* mRNA was confirmed by CLIP, and 3′-UTR luciferase reporter assay. *Kremen1* expression was down-regulated by miR-431 at the mRNA and protein levels. This may mean that miR-431 cleaves the mRNA of this gene rather than repressing its translation. To the best of our knowledge this is the first observation of direct mRNA target cleavage by miR-431. At the same time, our data do not exclude possibility that there is another miRNA or transcription factor that may regulate *Kremen1* too.

*Kremen1* was originally discovered as a transmembrane protein containing the kringle domain. Later reports confirmed that both *Kremen1* and its relative Kremen2 were high-affinity receptors for Dickkopf1 (Dkk1), the inhibitor of Wnt/β-catenin signaling (Mao et al., 2002). The canonical Wnt/β-catenin signaling is mediated by two receptor families, Frizzle protein and lipoprotein-receptor-related protein 5 and 6 (LRP5/6). *Kremen1* functionally cooperates with Dkk1 to form a ternary complex composed of *Kremen1*, Dkk1, and LRP5/6, and induces rapid endocytosis and removal of the Wnt receptor LRP5/6 from the cell membrane, which inhibits the transduction of Wnt/β-catenin signaling. Wnt/β-catenin signaling plays a vital role in diverse developmental and physiological processes, including cell-fate determination, tissue patterning, and stem cell regulation (Diep et al., 2004). Wnt/β-catenin signaling pathway also contributes to adult neurogenesis. Blocking Wnt signaling abolishes neurogenesis in adult hippocampal progenitor cells *in vitro* and suppresses neurogenesis *in vivo *(Lie et al., 2005). With ectopic expression of Dkk1, canonical Wnt/β-catenin signaling is markedly reduced in both the hippocampus and cortex (Solberg et al., 2008).

Studies have also established a role for Wnt signaling in regulating synaptic plasticity and axonal growth (Hall et al., 2000; Wang et al., 2006; Budnik and Salinas, 2011). Wnt signaling regulates axon terminal remodeling (Budnik and Salinas, 2011), formation of growth cones and lamellipodia (Hall et al., 2000), microtubules organization (Purro et al., 2008), and synaptic assembly (Ahmad-Annuar et al., 2006). Loss- and gain-of-function studies in animal models demonstrated that loss of Wnt7a results in a strong deficit in the accumulation of synaptic markers at the cell synapses (Ahmad-Annuar et al., 2006). In contrast, in cultured mouse cerebellar granule cells, Wnt7a increased neurite elongation and branching as well as the expression of synaptic markers (Lucas and Salinas, 1997). Likewise, targeted disruption of Wnt receptor genes in mice produced severe defects in axon growth and guidance, resulting in a loss of thalamocortical, nigrostriatal tracts, and the anterior commissure (Wang et al., 2002, 2006). Moreover, SCI induced a time-dependent increase in Wnt expression, phosphorylation of Wnt receptors, and activity of β-catenin protein. Thus, the activation of the Wnt pathway after SCI suggests the involvement of Wnt pathway in nerve regeneration (Fernandez-Martos et al., 2011).

These abundant evidences from studies in animal models, cell and organ culture firmly established an important role of Wnt signaling in neurite outgrowth and axonal guidance. The function of Wnt signaling could potentially link our observation on increased miR-431 and decreased *Kremen1* expression to the enhanced axonal outgrowth. In our study, *Kremen1* loss-of-function produced an increase in axon outgrowth mimicking the effect of miR-431 gain-of-function but did not increase branching. The axon elongation is a critical factor for axon regeneration. The excessive branching can be detrimental to axon regeneration, especially in the PNS. Evidence suggests that axonal elongation and branching are differentially regulated in hippocampal neurons (Pujol et al., 2005).

Taken together, our studies identified miR-431 as an endogenous, injury-regulated inhibitor of *Kremen1*, which promotes regenerative axon growth in adult sensory neurons. Further studies are necessary to fully define the role of miR-431 in axonal regeneration. These findings may not only contribute to our understanding of fundamental biological process, but also could have important implication for improving the therapeutic strategies for nerve injury.

## Conflict of Interest Statement

The authors declare that the research was conducted in the absence of any commercial or financial relationships that could be construed as a potential conflict of interest.

## Footnotes

1 , ,
